# Quality and Antioxidant Properties of Cold-Pressed Oil from Blanched and Microwave-Pretreated Pomegranate Seed

**DOI:** 10.3390/foods10040712

**Published:** 2021-03-26

**Authors:** Tafadzwa Kaseke, Umezuruike Linus Opara, Olaniyi Amos Fawole

**Affiliations:** 1 Department of Food Science, Faculty of AgriSciences, Stellenbosch University, Private Bag X1, Stellenbosch 7602, South Africa; tafakaseqe@gmail.com; 2 Faculty of AgriSciences, Africa Institute for Postharvest Technology, South African Research Chair in Postharvest Technology, Stellenbosch University, Private Bag X1, Stellenbosch 7602, South Africa; 3 Department of Botany and Plant Biotechnology, Faculty of Science, University of Johannesburg, P.O. Box 524, Johannesburg 2006, South Africa

**Keywords:** pomegranate seed, oil, pretreatment, cold pressing, total phenolic content, antiradical activity

## Abstract

The present research studied the influence of blanching and microwave pretreatment of seeds on the quality of pomegranate seed oil (PSO) extracted by cold pressing. Pomegranate seeds (cv. Acco) were independently blanched (95 ± 2 °C/3 min) and microwave heated (261 W/102 s) before cold pressing. The quality of the extracted oil was evaluated with respect to oxidation indices, refractive index, yellowness index, total carotenoids content, total phenolic content, flavor compounds, fatty acid composition, and 2.2-diphenyl-1-picryl hydrazyl (DPPH) and 2.2-azino-bis (3-ethylbenzothiazoline-6-sulfonic acid) (ABTS) radical scavenging capacity. Blanching and microwave pretreatments of seeds before pressing enhanced oil yield, total phenolic content, flavor compounds, and DPPH and ABTS radical scavenging capacity. Although the levels of oxidation indices, including the peroxide value, free fatty acids, acid value, ρ-anisidine value, and total oxidation value, also increased, and the oil quality conformed to the requirements of the Codex Alimentarius Commission (CODEX STAN 19-1981) standard for cold-pressed vegetable oils. On the other hand, blanching and microwave heating of seeds decreased the pomegranate seed oil’s yellowness index, whilst the refractive index was not significantly (*p* > 0.05) affected. Even though both blanching and microwave pretreatment of seeds added value to the cold-pressed PSO, the oil extracted from blanched seeds exhibited lower oxidation indices. Regarding fatty acids, microwave pretreatment of seeds before cold pressing significantly increased palmitic acid, oleic acid, and linoleic acid, whilst it decreased the level of punicic acid. On the contrary, blanching of seeds did not significantly affect the fatty acid composition of PSO, indicating that the nutritional quality of the oil was not significantly affected. Therefore, blanching of seeds is an appropriate and valuable step that could be incorporated into the mechanical processing of PSO.

## 1. Introduction

Pomegranates (*Punica granatum* L.) are consumed as fresh fruits and processed into products such as jam, juice, jelly, wine, and dried snacks [1]. In addition to increased production volumes, the inconvenience associated with fresh pomegranate consumption due to the fruit complexity has promoted the fruit’s processing into these ready to eat and convenient products [2]. Moreover, the consumption of the fruit is related to its medicinal properties. The fruit’s pharmacological value can be traced back to ancient times when the fruit was used as a traditional medicine to treat different ailments [3].

The literature has shown that every part of the fruit contains compounds with health benefits. The juice and peels contain punicalagins, hydrolyzable tannins, anthocyanins, and ellagic acid [4]. Pomegranate seeds, one of the waste products from the processing of the fruit, serve as a rich source of oil (12–20%) high in tocopherols, polyphenols, sterols, and punicic acid [5]. It has been demonstrated that these bioactive phytochemicals are implicated in pomegranate seed oil’s chemopreventive activities such as anti-mutagenicity, antihypertension, antioxidative potential, and reduction in liver injury [6]. In line with this, pomegranate seed can be considered for value-added products. Further, processing the seeds into specialty oil is a profitable alternative to managing the postharvest waste from pomegranate fruit processing. Pomegranate seed oil can be extracted from the seeds using various techniques such as cold pressing and solvent, supercritical carbon dioxide, and ultrasound-assisted aqueous enzymatic extraction [7,8]. Prior research has indicated that the extraction technique is a major determinant of seed oil quality [9].

Seed oil extraction by cold pressing is the most preferred by processors and consumers because of the low production costs and high concentration of bioactive compounds such as essential fatty acids, tocopherols, phenols, carotenoids, and phytosterols in the oil [10]. The retention of antioxidant compounds may provide cold-pressed oils with acceptable oxidative stability and better health properties [11]. Cold-pressed oils are obtained mechanically using either a hydraulic or screw press without the application of heat, solvents, or chemical treatments, which makes the process environmentally friendly and the extracted oil safer for human consumption [12]. Therefore, there is a growing demand for cold-pressed oil, such as cold-pressed pomegranate seed oil. The maximum temperature of cold-pressed oil should not exceed 50 °C [13,14]. The cold-pressed oil may be physically purified through filtration, sedimentation, or centrifugation processes, which do not degrade the oil quality [10].

Despite the many advantages of cold pressing, the low-cost and sustainable oil extraction technique suffers from low oil yield due to a significant amount of oil that remains trapped in the pressed meal, which has hindered its development and commercial viability [15]. Nonetheless, this may be improved by blanching or microwave heating the oil-bearing seeds before pressing. According to Kaseke et al. [9], blanching seeds improved the pomegranate seed oil yield and bioactive compounds recovery with ethanol. Moreover, seed blanching is a novel technique that presents a sustainable strategy capable of improving seed oil quality whilst significantly reducing the oil extraction time and energy consumption during cold pressing [16]. Blanching significantly changes the seed matrix’s structural integrity by disintegrating the cell walls and membranes, which may enhance the extractability of the intracellular material by cold pressing [17]. Nevertheless, microwave pretreatment is the commonly used technique to improve oil yield and bioactive compounds recovery in cold-pressed oils [18,19,20,21], due to its uniform energy delivery, high thermal conductivity to the interior of the material, energy saving, and precise process control [22]. Although the influence of seed pretreatment on the oil recovery efficiency of mechanical pressing has been studied, comparative studies on seed pretreatment techniques’ potential to improve the quality of cold-pressed oil are limited.

In this regard, the current study aimed to investigate the effect of blanching and microwave heating pomegranate seeds on the quality and functional properties of oil extracted by cold pressing.

## 2. Materials and Methods

### 2.1. Plant Material

‘Acco’ pomegranates were harvested in April during the 2019 season from Blydeverwacht Farm (33°48′0″S, 19°53′0″E) in Western Cape Province, South Africa, at the commercial maturity stage (total soluble solids: 14.02–16.61°Brix). The seeds were separated from the peels, membranes, and juice before they were thoroughly cleaned with tap water.

### 2.2. Sample Preparation and Pretreatments

#### 2.2.1. Blanching

Freshly extracted and clean pomegranate seeds (PS) were blanched in a water bath (Scientific, South Africa) at 95 ± 2 °C for 3 min [9]. After blanching, samples were cooled promptly in an ice water bath, drained off, and then oven dried at 55 ± 2 °C to 10% (*w/w*) moisture content. The thermogravimetric technique was applied to measure the moisture content using a moisture analyzer set at 100 °C (KERN, DBS60-3, Balingen, Germany).

#### 2.2.2. Microwave Pretreatment

Fifty grams of oven-dried PS were uniformly spread in a glass Petri dish (190 mm in diameter) inside a calibrated domestic microwave oven (Model: DMO 351, Defy Appliances, Cape Town, South Africa) with a nominal power of 900 W. The microwave oven was calibrated following the method described by Rekas et al. [23]. The samples were microwave heated at 2450 MHz and 261 W for 102 s [24]. The microwave-heated seeds were cooled at ambient temperature (25–27 °C) and thoroughly mixed to uniform samples. The moisture content of the seeds after microwave heating was adjusted to 10%.

### 2.3. Cold Pressing

PS (250 g) were pressed using a single-screw press (Farmet UNO, Ceska Skalice, Czech Republic) equipped with a 10 mm diameter die. The capacity of the expeller press is about 8–12 kg seed/h. The press head was heated to 60 ± 5 °C before oil pressing using a removable heating element, and the temperature of the outflowing oil was 50 ± 5 °C. Temperature was measured using a type-K thermocouple connected to a digital temperature sensor (KIMO Instruments, Wilmington, NC, USA). The pressed oil was centrifuged at 4000 rpm for 15 min (Centrifuge 5810R, Eppendorf, Horsholm, Germany) to remove solid particles. Pomegranate seed oil (PSO) extraction yield was defined as gram per hundred gram of pomegranate seed (g/100 g seed). The oil samples were packed in brown bottles and stored at −20 °C before further analyses to minimize oxidation.

### 2.4. Determination of Pomegranate Seed Oil Quality Indices

#### 2.4.1. Yellowness and Refractive Index 

The refractive index was evaluated at 25 °C using a calibrated Abbe 5 refractometer (Bellingham + Stanley, Kent, United Kingdom). The yellowness index (YI) was calculated from the PSO color properties, including lightness (L*) and yellowness (b*) values, which were measured using a calibrated Chroma meter CR-410 (Konica Minolta, INC, Tokyo, Japan).

(1) $$\mathrm{YI}=\frac{142.86b^{*}}{L^{*}}$$

#### 2.4.2. Oxidation Indices

Free fatty acids (FFA) and acid value (AV) were measured following the AOCS standard [25]. The modified ferrous oxidation-xylenol orange (FOX) method was used to determine peroxide value (PV) [26]. ρ-Anisidine value (AnV) was determined according to [25]. Total oxidation (TOTOX) value was calculated from the PV and AnV using Equation (2).

(2) TOTOX=2PV+AnV

### 2.5. Determination of Bioactive Compounds and Antiradical Activity

#### 2.5.1. Total Carotenoids Content and Total Phenolic Content

The method described by Ranjith et al. [27] was used to determine total carotenoids content (TCC). PSO (0.2 g) was dissolved in hexane (5 mL) and 0.5 mL sodium chloride (NaCl) (0.5%, *w/w*) and thoroughly vortexed before being centrifuged (Centrifuge 5810R, Eppendorf, Horsholm, Germany) at 4000 rpm for 5 min. The absorbance was measured at 460 nm using a UV spectrophotometer (Spectrum Instruments, United Scientific, Cape Town, South Africa), and the results were expressed as mgβ-carotene/g of PSO. The Folin–Ciocalteu method was applied to evaluate the total phenolic content (TPC) [28]. Briefly, 200 µL of PSO methanol extracts, 250 µL of the Folin–Ciocalteau reagent, 750 µL of 2% (*w/v*) sodium carbonate, and 3 mL of distilled water were sequentially mixed, and the mixtures were vortexed and incubated in the dark for 40 min. The absorbances were measured at 760 nm using a UV spectrophotometer (Spectrum Instruments, United Scientific, Cape Town, South Africa), and the results were reported as milligram gallic acid equivalent per g PSO (mg GAE/g PSO).

#### 2.5.2. Antiradical Activity

The PSO antiradical activity was evaluated using 2.2-azino-bis (3-ethylbenzothiazoline-6-sulfonic acid) (ABTS) and 2.2-diphenyl-1-picryl hydrazyl (DPPH) assays. Briefly, ABTS radical cation (ABTS^+^) stock solution, prepared by mixing equal volumes of 2.2-azino-bis (3-ethylbenzothiazoline-6-sulfonic acid) (ABTS) solution (7.4 mM) and potassium persulfate solution (2.6 mM), was kept in the dark for 12–16 h. The absorbance was adjusted to 0.7 ± 0.02 at 750 nm after the incubation period, using 80% (*v/v*) methanol [29]. Three hundred microliters of methanol PSO extracts were mixed with 300 µL of the freshly prepared ABTS^+^ solution and the samples were incubated for 10 min in the dark. The absorbances of the samples were measured at 750 nm using a microplate reader (Thermo Fisher Scientific, Shanghai, China). The results were reported as mmol Trolox/g of PSO.

The method described by Siano et al. [30] was used to determine the DPPH radical scavenging capacity of PSO. Aliquots of 200 µL of methanol PSO extracts were added to 2.5 mL of 0.04% (*w/v*) DPPH in 80% (*v/v*) methanol and vortexed before incubation in the dark for 60 min. The absorbance was measured using a UV spectrophotometer (Spectrum Instruments, United Scientific, Cape Town, South Africa) at 517 nm. Results were expressed as mmol Trolox/g of PSO.

### 2.6. Fatty Acid Composition

The gas chromatography-mass spectrometry (GC-MS) method was used to determine the PSO fatty acid composition [31]. PSO (0.1 g), 2.0 mL hexane, 50 µL heptadecanoic acid (1000 ppm, internal standard), and 1.0 mL of 20% (*v/v*) H_2_SO_4_ in methanol were sequentially mixed, vortexed, and incubated at 80 °C for 1 h in an oven. To the cooled samples, 3 mL of saturated NaCl was added, and the mixture was further vortexed before centrifugation (Centrifuge 5810R, Eppendorf, Horsholm, Germany) at 4000 rpm for 3 min. The supernatant (hexane extract) was analyzed using a gas chromatograph (6890N, Agilent Technologies, Palo Alto, CA, USA) coupled to a flame ionization detector (FID). The fatty acid methyl esters were separated on a polar RT-2560 (100 m, 0.25 mm ID, 0.20 µm film thickness) (Restek, Bellefonte, PA, USA) capillary column and helium (1 mL/min) was used as the carrier gas. The sample (1µL) was injected in a 5:1 split ratio and at 240 °C. The oven temperature was programmed as 60 °C/min and increased to 120 °C at a rate of 8 °C/min, then to 245 °C at 1.5 °C/min, and finally to 250 °C at 20 °C/min for 2 min. Gas-chromatographic peaks of FAME were identified by comparison with a commercial mixture of standards, and the NIST library was used to identify the pomegranate seed oil fatty acids profiles. The relative content (%) of each fatty acid was calculated by dividing the peak area of each fatty acid by the total peak area of all the fatty acids identified.

### 2.7. Determination of Volatile Compounds

Volatile compounds were analyzed by HS-SPME-GC-MS [32]. One thousand microliters of oil samples was put in 20 mL SPME vials and 1 µL was injected into the SPME-GC-MS system. Separation was performed on a gas chromatograph (6890N, Agilent technologies network) coupled to an Agilent technologies inert XL EI/CI Mass Selective Detector (MSD) (5975B, Agilent Technologies Inc., Palo Alto, CA). The GC-MS system was coupled to a CTC Analytics PAL autosampler. Separation of the oil volatiles was performed on a ZBWaxPlus (30 m, 0.25 mm ID, 0.25 µm film thickness) capillary column. Helium was used as the carrier gas at a flow rate of 1 mL/min. The injector temperature was maintained at 250 °C. Injection was performed in splitless mode. The oven temperature was programmed as follows: 35 °C for 5 min, followed by a ramping rate of 5 °C/min until 50 °C and held for 3 min, ramped again at a rate of 5 °C/min until 120 °C and held for 3 min, and finally ramped up to 240 °C at a rate of 10 °C/min for 3 min. The MSD was operated in a full scan mode, and the source and quad temperatures were maintained at 230 °C and 150 °C, respectively. The transfer line temperature was maintained at 250 °C. The mass spectrometer was operated under electron impact (EI) mode at an ionization energy of 70 eV, scanning from 25 to 650 m/z. Compound identification was based on mass spectral data of samples with the standard NIST and Wiley Library and with the comparison of retention indices. The relative content (%) of each volatile compound was calculated by dividing the peak area of each component by the total peak area of all the compounds identified.

### 2.8. Statistical Analysis

The results of all the studied variables are presented as mean ± SD (standard deviation). One-way analysis of variance (ANOVA) was carried out using Statistica software (Statistical v13, TIBC, Palo Alto, CA 94304, USA) and the mean values were separated according to Duncan’s multiple range test. Graphical presentations were made using Microsoft Excel (Version: 16.0.13029.20344, Microsoft Cooperation, Washington, DC, USA).

## 3. Results and Discussion

### 3.1. Oil Yield

**Figure 1:**
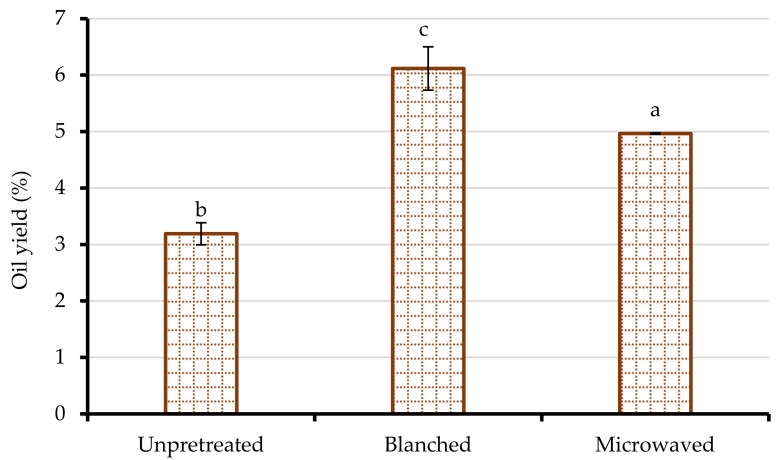
Oil yield of cold-pressed pomegranate seed oil from unpretreated, blanched (95 ± 2 °C/3 min), and microwave-heated (261 W for 102 s) seeds. Columns followed by different letters are significantly different (*p* < 0.05) according to Duncan’s multiple range test. Vertical bars indicate the standard deviation of the mean.

Oil yield is an essential factor in maximizing the gross income for seed oil processers. In this regard, pretreatment of the oleaginous material is crucial in promoting oil release from the seed matrix. The results in Figure 1 show that blanching and microwave heating of seeds enhanced the PSO yield (55 and 91%, respectively), with blanched seeds exhibiting a significantly higher oil yield (6.12%) than microwave-heated seeds (4.97%). The initial yield of PSO from unpretreated seeds was 3.20%. The finding that blanching and microwave heating of seeds significantly improved the PSO yield could be attributed to altering the pomegranate seed cellular structures, which increased the permeability of the cell walls and mass transfer of lipids during pressing [9,19]. Prior research has also reported significant enhancement of cold press oil extraction efficiency by thermal seed pretreatment [18,33,34]. For instance, seed microwave pretreatment doubled the yield of cold-pressed black cumin seed oil [35]. In a recent study, Lee et al. [36] observed a 2.3- to 2.4-fold increase in cold-pressed perilla seed oil yield after steam pretreatment of the seeds. On the other hand, in the absence of seed pretreatment, which provides cell disintegration, the permeability of the pomegranate seed to oil could have been limited, hence the lower oil yield from unpretreated seeds (Figure 1) [37]. The oil yield in the current study is 1.1- to 2.2-fold lower than the one reported by Khoddami et al. [7], a fact that could be explained by differences in seed variety, moisture content, oil press equipment, fruit maturity, and geographical location, among other factors. 

### 3.2. Yellowness and Refractive Index

**Figure 2:**
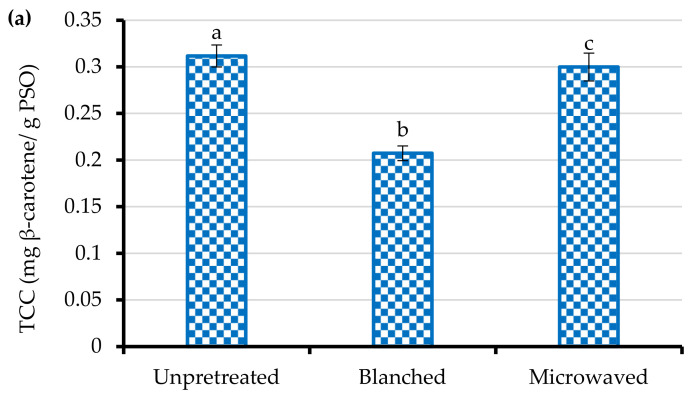

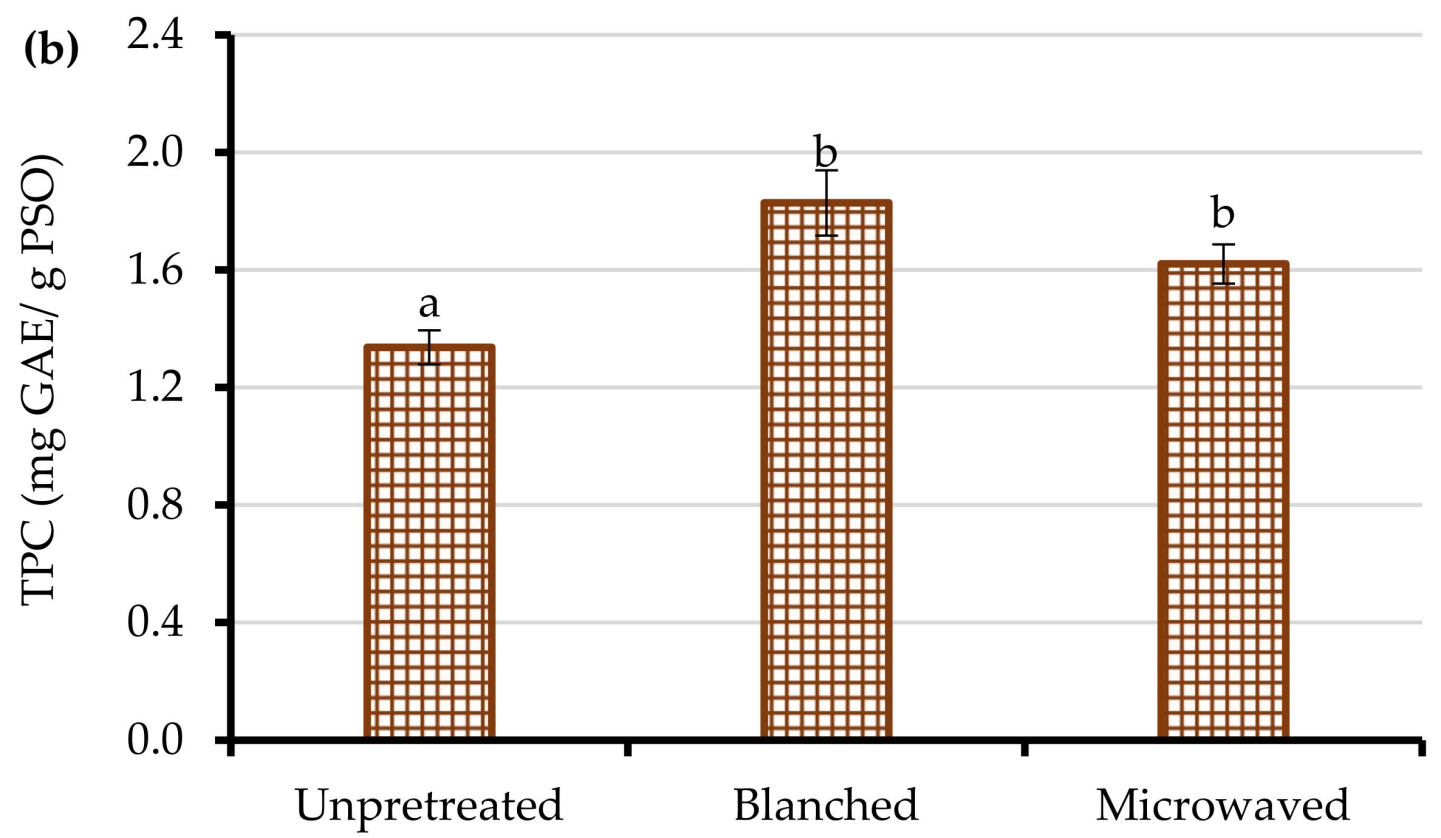
(**a**) Total carotenoids content (TCC) and (**b**) total phenolic content (TPC) of cold-pressed pomegranate seed oil (PSO) from unpretreated, blanched (95 ± 2 °C/3 min), and microwave-heated (261 W for 102 s) seeds. Columns followed by different letters are significantly different (*p* < 0.05) according to Duncan’s multiple range test. Vertical bars indicate the standard deviation of the mean.

The color and appearance of foods, including seed oil, constitute the first set of sensory attributes and therefore affect the consumer perception of quality. The color of food may be attributed to natural pigments or biochemical or chemical products developed during processing such as seed thermal pretreatment [38]. The effect of processing on food product color can be determined by measuring the YI. Table 1 depicts the changes in the PSO yellowness index due to seed blanching and microwave heating. Blanching and microwave heating of PS significantly decreased the oil YI by 7%. With respect to PSO from blanched seeds, the decrease in the YI could be ascribed to the reduction in total carotenoids content due to the conversion of trans-carotenoids, which are the usual configuration, to cis-isomers, hence decreasing the oil yellowness (Figure 2a) [39]. According to Kha et al. [40], the extensive conjugated and trans-configured double bond system in carotenoids absorbs light in the visible region and provides foods such as seed oil with color. The pomegranate seed oil YI ranged from 37.30 to 40.21 and was lower than the one observed by Khoddami et al. [7] from cold-pressed oil (81.15–91.55) of different pomegranate cultivars. 

The refractive index is often applied to identify and characterize food materials, including seed oil. The relationships between RI and fatty acid chain length as well as the degree of unsaturation have been reported [41]. The refractive index of the oil did not significantly (*p* > 0.05) change after seed pretreatment, regardless of the significant change in fatty acid content after seed microwave pretreatment. In this sense, the interpretation of RI results in the present study should be made with caution. The RI narrowly ranged between 1.5194 and 1.5197, values typical of PSO and indicative of its high unsaturation (Table 1) [41]. These values agree with those reported by Costa et al. [42] (1.5091–1.5177) from cold-pressed PSO.

### 3.3. Peroxide Value, Free Fatty Acids, Acid Value, ρ-Anisidine, and Total Oxidation Value

The PV is used as the quantity measurement for peroxides, which are intermediate products of lipid oxidation. The PV test is a good way to determine the amount of primary oxidation products in freshly extracted seed oil. The PV of cold-pressed PSO from unpretreated seeds was relatively low (0.73 meqO_2_/kg PSO). After seed blanching and microwave heating, the PV significantly (*p* < 0.05) increased by 11 and 18%, although no significant differences were observed between the PV of oils extracted from microwave-heated and blanched seeds (Table 1). Nevertheless, the PVs (0.73–0.86 meqO_2_/kg PSO) from all oil samples were far below the level (15 meqO_2_/kg oil) established by the World Health Organization (WHO) under the Codex Alimentarius Commission, indicating that the oils were of good quality and acceptable at the international market [43]. The lower values of peroxides in the oil samples may result from the lower extraction temperatures during oil pressing.

Free fatty acids and the acid value may be used to indicate lipase activity in the seed oil [7]. In this sense, higher FFA and AV in seed oil indicate a higher magnitude of hydrolytic deterioration and lower-quality oil product. As shown in Table 1, PSO from blanched and microwave-heated seeds had a relatively higher FFA and AV than PSO from unpretreated seeds. The increase in FFA and AV after seed blanching and microwave heating ranged between 7 and 54%. According to the quality requirements as recommended by the Codex Alimentarius Commission, cold-pressed oils should have a maximum of 4.0 mg KOH/g oil of AV [43]. Regardless of the increase after seed blanching and microwave heating, the AVs (1.19–1.83 mg KOH/g PSO) were within the standardized requirements (Table 1). The FFA (0.60–0.92%) in the present study were lower than those reported in previous studies. For instance, Khoddami et al. [7] reported FFA values of cold-pressed PSO ranging from 0.65 to 1.39%, which were 1.1- to 1.5-fold higher than our results.

The ρ-anisidine value measures the aldehyde and ketonic breakdown products of peroxides. These secondary products of oxidation are responsible for the development of rancidity in oils and fats. As shown in Table 1, the AnV of PSO from unpretreated, blanched, and microwave-heated seeds were 1.97, 2.71, and 3.02, respectively, which were 6 to 7 times lower than those reported by Costa et al. [42]. The result that microwave heating of seeds significantly increased the AV of PSO by 53% while blanching had an insignificant effect on AV indicates the difference in the pretreatment methods’ mode of action. Despite the unavailability of an internationally recognized seed oil quality standard on AnV, there is a general agreement among researchers that for seed oil to be still acceptable, the AnV should be less than 10 [42,44].

The total oxidation value of PSO was determined using the PV and AnV values, representing the information for primary and secondary oxidation products. Therefore, the TOTOX value indicates both the oxidation history and further oxidation potential of the oils [45]. The changes in TOTOX values due to pomegranate seed pretreatment are shown in Table 1. The TOTOX value for PSO from unpretreated seeds was 3.42, which significantly increased to 4.33 and 4.74 after seed blanching and microwave heating, respectively. The results suggest that blanching and microwave heating of seeds could have promoted lipase enzyme activity and hydrolytic oxidation of the oil. The literature has reported increased activity of lipolytic enzymes on fat and oil in damaged cells [46].

### 3.4. Total Carotenoids Content, Total Phenolic Content, and Antiradical Activity

While the consumption of foods rich in carotenoids has been strongly linked to the reduction in incidences of diseases such as cancers, cardiovascular diseases, age-related macular degeneration, and cataracts, these thermolabile antioxidant compounds might be affected by processing [6]. According to Figure 2a, TCC significantly decreased (32%) after pomegranate seed blanching. Nevertheless, it was not significantly (*p* > 0.05) affected by seed microwave heating. The decrease in TCC after seed blanching could be explained by the breakdown of carotenoid molecules through isomerization and thermal oxidation [20]. These values were higher than TCC values reported in previous studies. For example, Costa et al. [42] reported TCC values ranging between 0.010 and 0.015 mg β-carotene/g PSO. Moreover, other studies failed to detect carotenoids in PSO [5]. The variation in the results could be due to dissimilarities in cultivars, fruit maturity, geographical location, and oil extraction process, among other factors [47]. It should also be highlighted that the absorbance in the spectrophotometric method might be increased by compounds other than carotenoids, which are active in the carotenoids’ spectral range (400–500 nm) [48]. The TCC from other fruit seeds such as passion fruit and sour cherry ranged between 0.01 and 1.20 mg β-carotene/g oil [49,50]. The large disparity in the TCC of oil from different fruit seeds could reflect differences in the sensitivity of the methods of analysis, and it is, therefore, suggested that TCC calculated from the sum of individual carotenoids could be more reliable.

The total phenolic contents of PSO from unpretreated, blanched, and microwave-heated seeds are presented in Figure 2b. Blanching and microwave heating of pomegranate seeds significantly improved the TPC of cold-pressed oil by 21 and 37%, respectively. The findings suggest that blanching and microwaving of seeds facilitated the dissociation of glycosylated and esterified phenolic compounds, enhancing the amount of free phenolic compounds available for extraction [51]. The results coincide with Mazaheri et al. [20] and Lee et al. [36], who also reported improvement in TPC of cold-pressed black cumin and perilla seed oils after seed microwave and steam pretreatments, respectively. The levels of TPC from blanched and microwaved seeds did not significantly differ (*p* > 0.05). Given the potential bioactivity of phenolic compounds and the possible application of PSO as a functional food, enhancement of TPC after seed pretreatment was a desirable development. While the study of Zaouay et al. [52] reported TPC ranging from 0.03 to 0.07 mgGAE/g PSO, the TPC values in the current study varied from 1.33 to 1.83 mgGAE/g PSO (Figure 2b). Among other factors, the observed variation could be due to the selective nature of solvent extraction towards the phenolic compounds, hence the lower TPC values compared to the cold-pressed oil. On the contrary, Khoddami et al. [7] cold pressed oil from three different pomegranate cultivars and reported TPC values ranging from 8.52 to 10.44 mgGAE/g PSO that were 5.7 to 6.4 times higher than our results. These dissimilarities highlight the importance of preharvest and processing factors consideration in PSO processing and quality.

**Figure 3:**
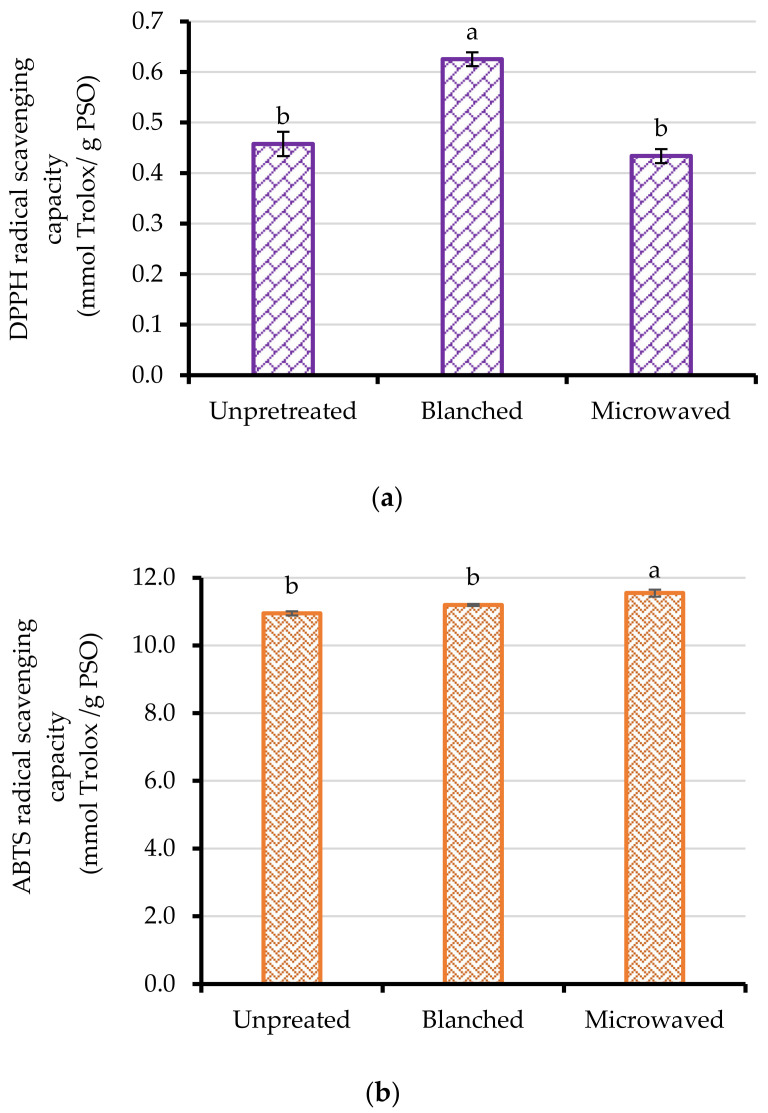
(**a**) DPPH and (**b**) ABTS radical scavenging capacity of cold-pressed pomegranate seed oil (PSO) from unpretreated, blanched (95 ± 2 °C/3 min), and microwave-heated (261 W for 102 s) seeds. Columns followed by different letters are significantly different (*p* < 0.05) according to Duncan’s multiple range test. Vertical bars indicate the standard deviation of the mean.

The antiradical radical activity of PSO was determined using the DPPH and ABTS assays. The DPPH radical scavenging activity of the cold-pressed PSO from unpretreated, blanched, and microwaved seeds is given in Figure 3a. While blanching seeds significantly improved the DPPH radical scavenging activity of the oil by 37%, microwave heating did not significantly (*p* > 0.05) change the DPPH radical scavenging activity of the cold-pressed PSO. Despite the insignificant effect of seed microwave pretreatment on the DPPH radical scavenging activity of the oil, previous studies on purslane and rape seed have shown increased DPPH radical scavenging activity in cold-pressed oil after seed microwave heating [53,54]. It is nevertheless noteworthy that seed physical and cellular structures that vary among different types of seeds and cultivars play a vital role in the efficiency of seed pretreatment, cold pressing, and recovery of the antioxidant compounds. Considering that antioxidant properties of oil have a major effect on its oxidative stability behavior, the PSO from blanched seeds might exhibit better stability and improved shelf life. The ABTS radical scavenging activity of the oil samples ranged between 10.95 and 11.55 mmol Trolox/g PSO, with oil from microwaved seeds exhibiting significantly higher ABTS scavenging activity than oil from blanched and unpretreated seeds (3 and 5%, respectively). The variation in the oil samples’ (microwaved and blanched seeds) DPPH and ABTS radicals scavenging suggests that the antioxidant compounds react differently with the different radicals, due to factors such as synergism [55]. The high ABTS scavenging activity (10.95–11.55 mmol Trolox/g PSO) in the present study could be attributed to the high levels of phenols in the cold-pressed oils and their synergistic effect with other antioxidant compounds such as tocopherols, which are also abundantly found in PSO [55,56].

### 3.5. Fatty Acid Composition

Fatty acid composition is one of the most critical quality characteristics of oilseeds, considering that the suitability of the oil for food, nutraceutical, or pharmaceutical applications may be governed by the type of fatty acids. Table 2 shows the fatty acid composition of cold-pressed PSO from unpretreated, blanched, and microwaved seeds. Chromatograms of FAMES for the treatments are presented in Supplementary Figure S1. Ten different types of fatty acids were identified in PSO, with palmitic acid, oleic acid, linoleic acid, and punicic acid being the primary fatty acids and representing 7.73–9.22%, 9.53–10.48%, 15.93–17.11%, and 54.12–58.32% of the total composition, respectively. Other fatty acids identified but in minor quantities (0.06–4.32%) were arachidic acid, stearic acid, heneicosanoic acid, docosanoic acid, docosenoic acid, and linolenic acid. Generally, thermal pretreatment of oilseeds may alter the fatty acids composition due to the sensitivity of polyunsaturated fatty acids [33]. While microwave heating of seeds significantly decreased punicic acid by 7%, blanching did not significantly (*p* > 0.05) affect the fatty acid. In a similar study, Ozcan et al. [21] observed a 14 and 11% decrease in punicic acid after pomegranate seed roasting (150 °C for 10 min) and microwave heating (750 W for 7.5 min), respectively. Considering that punicic acid is implicated in most PSO biochemical properties, the decrease in punicic acid after microwave heating of the seeds in the current study was not desirable. Although punicic acid has been reported in other seeds such as bitter gourd [6], pomegranate seed remains the major source of this bioactive lipid. Compared to the literature, the levels of punicic acid (54.12–58.32%) in the current study are comparable to those reported by Costa et al. [42] (55.24–60.62%) from cold-pressed PSO. Nevertheless, some previous studies on cold-pressed PSO reported values that were higher (75.23–78.23%) than in the present study [7,57]. The dissimilarities in the punicic acid content could be ascribed to variation in processing techniques and pomegranate cultivars, among other factors. Linoleic acid and γ-linolenic acid, essential fatty acids, significantly increased by 1.1- and 3.4-fold after microwaving the pomegranate seed, whilst the blanching of the seeds did not significantly change the respective fatty acids. This indicates differences in microwaving and blanching modes of action and their impact on the fatty acids. Owing to the absence of appropriate enzymes, the human body cannot synthesize these essential fatty acids, and therefore their maximum extraction from oilseeds is essential [58]. Oleic acid, the major monosaturated fatty acid in PSO, insignificantly varied from 9.68% to 9.53% and 10.48% after blanching and microwave heating the seeds, respectively (Table 2). Although the concentration of palmitic acid and arachidic acid, the main saturated fatty acids, increased between 1.4 and 19% and 42 and 43%, respectively, after seed blanching and microwave heating, the levels of stearic acid, heneicosanoic acid, and docosanoic acid were not significantly changed by seed pretreatment. The insignificant effect of seed thermal pretreatment on some fatty acids has also been reported in prior research [19].

Regarding total saturated fatty acids (SFA), blanching and microwave heating of seeds significantly increased the SFA by 9 and 18%, respectively. A 9% decrease in total monosaturated fatty acids (MUFA) was observed in PSO from blanched seeds, and this could be due to the significant decrease (7-fold) in docosenoic acid. No significant (*p* > 0.05) variation in MUFA of PSO pressed from microwaved seeds was observed. The total polyunsaturated fatty acids (PUFA) of oil from unpretreated seeds were 74.32% (Table 2). After seed microwave pretreatment, the level significantly decreased by 6%, whilst it insignificantly decreased in PSO from blanched seeds, indicating increased heat penetration and oxidation of polyunsaturated fatty acids during seed microwave heating (Table 1). The MUFA/PUFA index, which could be used as an indicator of the PSO stability to oxidation, among other factors [59], did not significantly vary after seed pretreatment. The finding implies that seed pretreatment did not affect the balance between the monosaturated and polyunsaturated fatty acids. However, the unsaturated fatty acids (UFA) to SFA index decreased after seed pretreatment, which could be explained by the significant increase in SFA after seed pretreatment.

### 3.6. Volatile Compounds

The results of volatile compounds of cold-pressed PSO from unpretreated, blanched, and microwave-heated seeds are presented in Table 3. A typical chromatogram of volatiles from the investigated pomegranate seed oil is presented in Supplementary Figure S2. Volatile compounds that can be perceived by humans have a greater influence on PSO flavor. These are the primary volatile flavor substances and constitute the characteristic flavor of PSO. The PSO samples showed varied volatile compounds belonging to the following chemical classes: alcohols, aldehydes, ketones, esters, carboxylic acids, and hydrocarbons. The groups of volatile compounds were comparable to the findings of Costa et al. [42] and Dun et al. [60] from cold-pressed pomegranate seed and peanut oils, respectively. 

Esters, which are derived from the esterification of free fatty acids and alcohols, occur naturally in many fruits and enhance their flavors. Pentyl pentanoate, the only ester observed in the oil samples, was significantly higher in oil from blanched and microwaved seeds (10- and 1.5-fold, respectively) than in unpretreated seeds, suggesting that blanching and microwaving the seeds can enhance the oil flavor [61]. Ren et al. [62] also reported the enhancement of ester compounds in rapeseed oil after microwave pretreatment of the seeds. In addition, blanching and microwave heating of seeds may induce heterocyclic compounds through the Maillard reaction, which enhances the positive flavors. Furan and its derivatives belong to heterocyclic compounds and correlate with the flavor of foods [62]. In the present study, 2.5-dimethyltetrahydrofuran was significantly higher (69%) in PSO from microwaved seeds when compared to unpretreated seeds.

Moreover, other furans including 2-pentylfuran and 2-butylfuran were only detected in oil from blanched and microwaved seeds. This phenomenon indicates that the flavor of PSO may be improved by blanching and microwave pretreatment of seeds. Pentanol, the primary alcohol observed, was 25 to 27% higher in PSO from blanched seeds than microwave-heated and unpretreated seeds (Table 3). Likewise, butanol and cycloheptanol manifested higher in PSO extracts from blanched than microwaved and unpretreated seeds. Other alcohol compounds observed in lower concentrations such as ethanol and octanol were not significantly affected by seed blanching and microwave heating. Alcohols have also been reported in previous studies as important contributors to seed oil flavor [63].

Among the aldehydes, pentanal was the major compound observed in the cold-pressed PSO and was significantly higher in PSO extracts from blanched and microwave-heated seeds than unpretreated seeds. Pentanal is characterized by a nutty and fruity flavor and has been naturally found in other seed oils such as sesame, olive, and peanut [63]. Other compounds including hexanal, 3-methylbutanal, 2-heptenal, and nonanal were also significantly higher in oil extracts from blanched and microwaved pomegranate seeds (Table 2). Aldehydes in seed oil are primarily produced either through the lipoxygenase pathway during oilseed cell fragmentation or automatic oxidation of the oil during production [64]. Hexanal is a typical oxidation volatile and has been commonly used as a quality indicator for lipid oxidation in seed oils. It is characterized by green, oily, and fruity odors [60]. Its level has been positively correlated with rancid taste. As shown in Table 3, blanching and microwave pretreatment of seeds may promote the oxidative degradation of the oil. Our results, therefore, indicate higher oxidation liability of oil from pretreated pomegranate seeds compared with unpretreated seeds. The PV, AV, and AnV results found in this study support these findings (Table 1). While 2-propanone did not significantly differ in all oil samples, other ketones such as 5-butyltetrahydro-2-furanone and 5-butyl-5H-furan-2-one were only detected in oil from pretreated seeds. Saturated fatty acids including hexanoic acid, acetic acid, pentanoic acid, formic acid, and butanoic acid did not significantly (*p* > 0.05) vary among the PSO samples. These free fatty acids, which are linked to sour and pungent sensations synonymous with sensory defects, could have been produced from the oxidation of their respective aldehydes [65]. It can be stated that, although seed blanching and microwave heating may augment the positive flavor of cold-pressed PSO, they may also promote the development of undesirable flavors.

## 4. Conclusions

In the current study, the effect of blanching and microwave pretreatment of seeds on the quality of cold-pressed PSO was investigated. Blanching and microwave pretreatment of seeds prior to pressing improved oil yield, total phenolic content, flavor compounds, and DPPH and ABTS radical scavenging capacity. The findings are desirable to pomegranate seed oil processors and consumers along the value chain, given that cold pressing is also a greener and safer technology compared to the use of solvents such as hexane. The levels of oxidation indices including the peroxide value, free fatty acids, acid value, ρ-anisidine value, and total oxidation value also increased. Nevertheless, the oil quality conformed to the requirements of the Codex Alimentarius Commission standard (CODEX STAN 19-1981) on cold-pressed vegetable oils.

On the other hand, blanching and microwave heating of seeds decreased the pomegranate seed oil’s yellowness index, whilst the refractive index was not significantly affected. Although both blanching and microwave pretreatment of seeds added value to the cold-pressed PSO, the oil extracted from blanched seeds exhibited lower oxidation indices. The finding affirms that the processing technique is one of the important seed oil quality determinants. Microwave pretreatment of seeds before cold pressing significantly increased palmitic acid, oleic acid, and linoleic acid, whilst it decreased the level of punicic acid, highlighting increased heat penetration and oxidation of the conjugated fatty acid. On the contrary, blanching of seeds did not significantly affect the fatty acid composition of PSO, an indication that the nutritional quality of the oil was not significantly affected. In conclusion, blanching of seeds is a practical step that could be incorporated into mechanical production of PSO.

## Figures and Tables

**Table 1:** Physicochemical properties of cold-pressed pomegranate seed oil (PSO) from unpretreated, blanched (95 ± 2 °C/3 min), and microwave-heated (261 W for 102 s) seeds.

Treatment	RI	YI	FFA	AV	PV	AnV	TOTOX
Unpretreated	1.5197 ± 0.00 ^a^	40.21 ± 0.03 ^a^	0.60 ± 0.04 ^b^	1.19 ± 0.09 ^b^	0.73 ± 0.02 ^a^	1.97 ± 0.15 ^b^	3.42 ± 0.14 ^a^
Blanched	1.5194 ± 0.00 ^a^	37.30 ± 0.08 ^b^	0.64 ± 0.10 ^ab^	1.28 ± 0.19 ^ab^	0.81 ± 0.01 ^b^	2.71 ± 0.43 ^ab^	4.33 ± 0.05 ^c^
Microwaved	1.5195 ± 0.00 ^a^	37.48 ± 0.09 ^b^	0.92 ± 0.09 ^a^	1.83 ± 0.18 ^a^	0.86 ± 0.02 ^b^	3.02 ± 0.08 ^a^	4.74 ± 0.09 ^b^

Means ± standard deviation of analysis (*n* = 3). Different superscript letters in the same column indicate significant difference (*p* < 0.05) according to Duncan’s multiple range test. RI = refractive index (25 °C), YI = yellowness index, FFA = free fatty acid as punicic acid (%), AV = acid value (mg KOH/g PSO), PV = peroxide value (meqO_2_/kg PSO, meqO_2_/kg = milli-equivalents of active oxygen per kg), AnV = ρ-anisidine value, TOTOX = total oxidation value.

**Table 2:** Fatty acid composition of cold-pressed pomegranate seed oil from unpretreated, blanched (95 ± 2 °C/3 min), and microwave-heated (261 W/102 s) seeds.

Treatment	Unpretreated	Blanched	Microwaved
**SFA**			
Palmitic (C16:0)	7.73 ± 0.30 ^b^	7.84 ± 0.14 ^b^	9.22 ± 0.22 ^a^
Stearic (C18:0)	2.53 ± 0.04 ^a^	2.53 ± 0.05 ^a^	2.64 ± 0.05 ^a^
Arachidic (20:0)	3.02 ± 0.16 ^b^	4.32 ± 0.20 ^a^	4.28 ± 0.18 ^a^
Heneicosanoic acid (C21:0)	1.04 ± 0.21 ^a^	1.14 ± 0.10 ^a^	0.88 ± 0.07 ^a^
Docosanoic acid (C22:0)	0.77 ± 0.11 ^a^	0.61 ± 0.04 ^a^	0.80 ± 0.02 ^a^
∑SFA	15.09 ± 0.50 ^b^	16.44 ± 0.19 ^a^	17.81 ± 0.30 ^c^
**MUFA**			
Oleic (C18:1 *n*-9 *cis*)	9.68 ± 0.12 ^b^	9.53 ± 0.24 ^b^	10.48 ± 0.17 ^a^
Docosenoic acid (C22:1)	0.92 ± 0.24 ^a^	0.13 ± 0.01 ^b^	0.25 ± 0.05 ^b^
∑MUFA	10.60 ± 0.15 ^a^	9.65 ± 0.25 ^b^	10.72 ± 0.22 ^a^
**PUFA**			
Linoleic (C18:2 *n*-6 *cis*)	15.93 ± 0.49 ^b^	16.00 ± 0.23 ^ab^	17.11 ± 0.18 ^a^
γ-Linolenic (C18:3 *n*-6)	0.07 ± 0.01 ^b^	0.06 ± 0.00 ^b^	0.24 ± 0.00 ^a^
Punicic (*cis*-9 *trans*-11 *cis*-13 C18:3)	58.32 ± 0.87 ^a^	57.85 ± 0.62 ^a^	54.12 ± 0.36 ^b^
∑PUFA	74.32 ± 0.50 ^a^	73.91 ± 0.41 ^a^	71.47 ± 0.52 ^b^
∑MUFA/∑PUFA index	0.14 ± 0.002 ^ab^	0.13 ± 0.004 ^b^	0.15 ± 0.004 ^a^
∑UFA/∑SFA index	5.64 ± 0.22 ^a^	5.09 ± 0.07 ^b^	4.62 ± 0.09 ^b^

Values (mean ± SD, *n* = 3) in the same row and followed by different superscript letters are significantly different (*p* < 0.05) according to Duncan’s multiple range test, SFA = saturated fatty acids, MUFA = monounsaturated fatty acids, PUFA = polyunsaturated fatty acids, UFA = unsaturated fatty acids, ∑ = sum of SFA, MUFA, or PUFA.

**Table 3:** Volatile compounds of cold-pressed pomegranate seed oil from unpretreated, blanched (95 ± 2 °C/3 min), and microwave-heated (261 W/102 s) seeds.

Treatment	Unpretreated	Blanched	Microwaved
**Alcohols**			
Cycloheptanol	0.77 ± 0.05 ^b^	1.58 ± 0.08 ^a^	1.32 ± 0.11 ^a^
Ethanol	1.84 ± 0.30 ^a^	1.74 ± 0.11 ^a^	1.26 ± 0.08 ^a^
Pentanol	10.82 ± 0.76 ^b^	13.70 ± 0.84 ^a^	10.92 ± 0.42 ^b^
Hexanol	0.95 ± 0.10 ^b^	1.83 ± 0.14 ^a^	1.47 ± 0.17 ^a^
Butanol	0.43 ± 0.01 ^b^	0.59 ± 0.02 ^a^	0.47 ± 0.00 ^b^
Octanol	0.51 ± 0.02 ^a^	0.52 ± 0.03 ^a^	0.43 ± 0.05 ^a^
Heptanol	0.80 ± 0.16 ^a^	1.13 ± 0.08 ^a^	ND
**Aldehydes**			
Hexanal	0.74 ± 0.01 ^b^	1.30 ± 0.09 ^a^	1.27 ± 0.08 ^a^
3-Methylbutanal	1.36 ± 0.08 ^b^	6.47 ± 0.96 ^a^	6.71 ± 0.55 ^a^
2-Heptenal	3.67 ± 0.65 ^b^	5.71 ± 0.11 ^a^	4.78 ± 0.35 ^ab^
Nonanal	0.78 ± 0.00 ^b^	1.04 ± 0.07 ^a^	1.05 ± 0.01 ^a^
2.4-*trans.trans*-Nonadienal	2.71 ± 0.34 ^a^	2.53 ± 0.16 ^a^	3.48 ± 0.50 ^a^
2.4 Nonadienal	3.05 ± 0.34 ^a^	2.72 ± 0.10 ^a^	2.97 ± 0.01 ^a^
Benzaldehyde	0.77 ± 0.08 ^a^	0.64 ± 0.03 ^a^	0.62 ± 0.04 ^a^
Pentanal	10.99 ± 0.09 ^a^	13.71 ± 0.83	11.87 ± 0.55 ^a^
Trans-2-hexenal	ND	0.59 ± 0.01 ^b^	0.50 ± 0.01 ^a^
Benzene acetaldehyde	ND	1.97 ± 0.28 ^b^	0.93 ± 0.17 ^a^
**Ketones**			
2-Propanone	6.00 ± 0.40 ^a^	7.01 ± 0.15 ^a^	6.44 ± 0.29 ^a^
5-Butyltetrahydro-2-furanone	0.22 ± 0.04	ND	ND
5-Butyl-5 H-furan-2-one	0.28 ± 0.03	ND	ND
**Carboxylic acids**			
Hexanoic acid	0.71 ± 0.03 ^a^	0.69 ± 0.03 ^a^	0.78 ± 0.04 ^a^
Acetic acid	3.23 ± 0.54 ^a^	3.46 ± 0.15 ^a^	4.11 ± 0.08 ^a^
Pentanoic acid	7.15 ± 1.19 ^a^	ND	6.02 ± 0.13 ^a^
Formic acid	ND	0.27 ± 0.01 ^a^	0.29 ± 0.02 ^a^
Butanoic acid	0.50 ± 0.02 ^b^	0.62 ± 0.02 ^a^	ND
**Esters**			
Pentyl pentanoate	0.69 ± 0.07 ^b^	6.81 ± 0.24 ^a^	1.05 ± 0.07 ^b^
**Furans**			
2-Pentylfuran	ND	0.21 ± 0.00	ND
2-Butylfuran	ND	0.36± 0.04 ^a^	0.37 ± 0.01 ^a^
2.5-Dimethyltetrahydrofuran	0.22 ± 0.02 ^a^	ND	0.35 ± 0.05 ^b^
**Hydrocarbons**			
*trans-alpha*-Bergamotene	0.74 ± 0.09 ^a^	0.32± 0.01 ^b^	0.36 ± 0.03 ^b^
2.3-Dimethyl-1-pentene	0.39 ± 0.06 ^a^	0.51 ± 0.05 ^a^	ND
Limonene	0.52 ± 0.09 ^b^	0.13 ± 0.01 ^a^	ND
**Others**			
Trichloromethane	2.43 ± 0.23 ^a^	2.35 ±0.44 ^a^	1.57 ± 0.09 ^a^

Means ± standard deviation of analysis (*n* = 3). Different superscript letters in the same row indicate significant difference (*p* < 0.05) according to Duncan’s multiple range test. ND = non-detected.

## Data Availability

The data presented in this study are available on request from the corresponding author. Some of the data is contained within Supplementary Materials.

## Supplementary Materials

The following are available online at https://www.mdpi.com/article/10.3390/foods10040712/s1, Figure S1: Chromatograms of FAMES of oil from unpretreated, blanched and microwave heated pomegranate seed, Figure S2: Typical chromatograms of volatiles from the pomegranate seed oil.
