# Supplementary material for: Quality and Antioxidant Properties of Cold-Pressed Oil from Blanched and Microwave-Pretreated Pomegranate Seed
Source: Foods. 2021 Mar 26;10(4):712. doi: 10.3390/foods10040712 (PMC8066041; doi:10.3390/foods10040712)
Supplement: Supplementary file 1 [file foods-10-00712-s001.pdf]

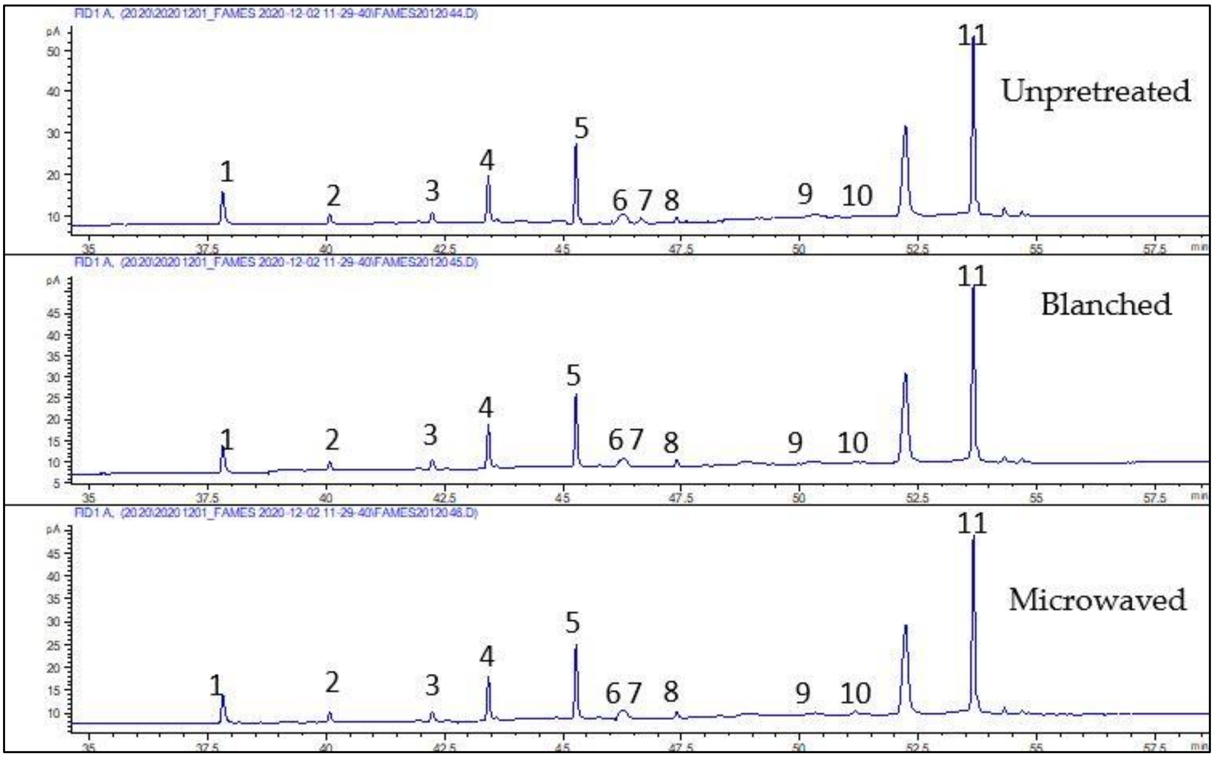

Figure 1. Chromatograms of FAMES of oil from unpretreated, blanched and microwave heated pomegranate seed. 1=C16:0 (37.8), 2=C17:0 (Internal Standard, 40.1), 3=C18:0 (40.2), 4= C18:1 n-9 *cis* (43.4), 5= C18:2 n-6 *cis* (45.3), 6= C20:0 (46.4), 7= C18:3 n-6, 8= C21:0 (48.2), 9= C22:0 (50.2), 10= C22:1 (51.0) 11=cis-9 trans-11 *cis*-13 C18:3 (54.0)

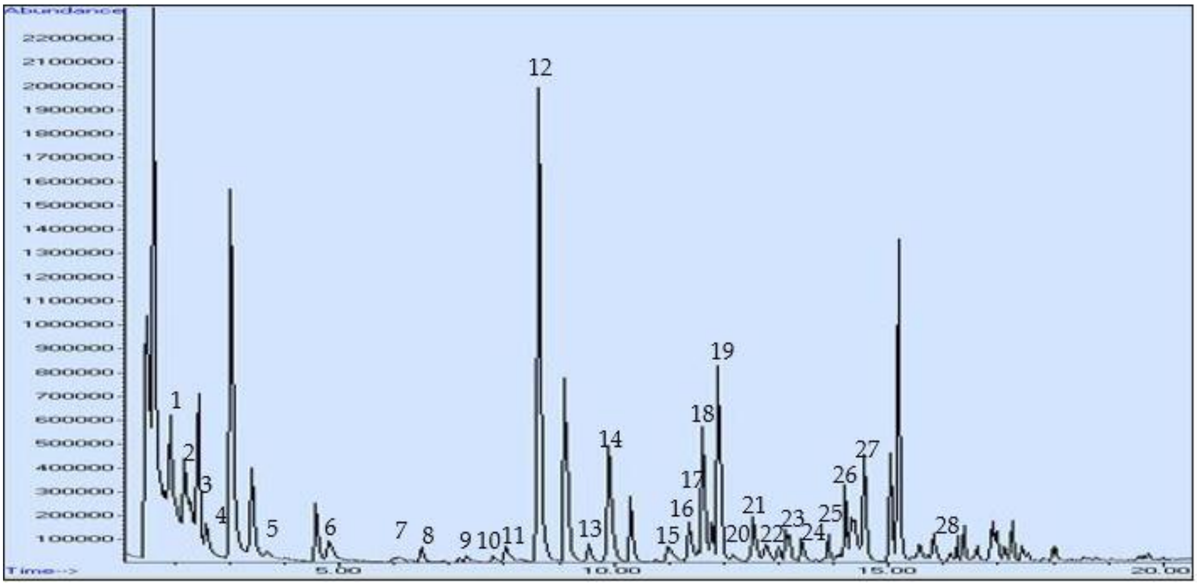

Figure 2. Typical chromatograms of volatiles from pomegranate seed oil. 1= 2-Propanone (1.9), 2= 3-Methylbutanal (2.4), 3= Ethanol (2.5), 4= Pentanal (3.0), 5= Trichloromethane (3.4), 6= Hexanal (4.8), 7= 5-Butyl-5H-furan-2-one (5.9), 8= Butanol (6.5), 9= Limonene (7.3), 10= Trans-2-hexenal (8.0), 11= 2-Pentylfuran (8.11), 12= Pentanol (8.6), 13= 2-Heptenal (9.9), 14= Hexanol (10.3), 15= Nonanal (11.0), 16= Pentanoic acid (11.4), 17= Pentyl pentanoate (11.4), 18= Acetic acid (11.6), 19= Heptanol (11.8), 20= Formic Acid (12.3), 21= Cycloheptanol (12.6), 22= Octanol (13.1), 23= 2.3-Dimethyl-1-pentene (13.1), 24= trans-alpha-Bergamotene (13.4), 25=Butanoic acid (14.0), 26= 2.5-Dimethyltetrahydrofuran (14.1), 27= 2.4 Nonadienal (14.4) and 28= Hexanoic acid (16.4)
